# The Pattern of Second Primary Tumours in Postmenopausal Women with Prior Breast Cancer in Western Romania: A Retrospective, Single-Institution Study

**DOI:** 10.3390/diagnostics11111957

**Published:** 2021-10-22

**Authors:** Cristina Marinela Oprean, Larisa Maria Badau, Robert-Alexandru Han, Teodora Hoinoiu, Gabriel-Mugur Dragomir, Daciana Grujic, Tiberiu Dragomir, Alis Dema

**Affiliations:** 1 ANAPATMOL Research Center, ‘Victor Babes’ University of Medicine and Pharmacy of Timisoara, 300041 Timisoara, Romania; cristina.oprean@oncohelp.ro (C.M.O.); dema.alis@umft.ro (A.D.); 2 Department of Oncology—ONCOHELP Hospital Timisoara, Ciprian Porumbescu Street, No. 59, 300239 Timisoara, Romania; larisa_badau@yahoo.com (L.M.B.); robert.han@oncohelp.ro (R.-A.H.); 3 Department of Oncology—ONCOMED Outpatient Unit Timisoara, Ciprian Porumbescu Street, No. 59, 300239 Timisoara, Romania; 4 Hygiene Discipline, “Victor Babeş” University of Medicine and Pharmacy, Eftimie Murgu Square Nr.2, 300041 Timişoara, Romania; 5 Department of Clinical Practical Skills, “Victor Babeş” University of Medicine and Pharmacy, Eftimie Murgu Square Nr.2, 300041 Timişoara, Romania; 6 Center for Advanced Research in Cardiovascular Pathology and Hemostaseology, “Victor Babeș” University of Medicine and Pharmacy, 300041 Timisoara, Romania; 7 Department of Teaching Training—POLYTEHNICAL, University of Timisoara, 300223 Timisoara, Romania; mugur.dragomir@upt.ro; 8 Department of Plastic and Reconstructive Surgery, “Victor Babeş” University of Medicine and Pharmacy, Eftimie Murgu Square Nr.2, 300041 Timişoara, Romania; dcalistru@yahoo.com; 9 Department V Internal Medicine, Discipline of Medical Semiology II, “Victor Babes” University of Medicine and Pharmacy, Eftimie Murgu Square Nr.2, 300041 Timisoara, Romania; dragomir.tiberiu@umft.ro

**Keywords:** second malignancies, breast cancer, second primary tumour sites, postmenopausal status, molecular subtype, luminal subtype

## Abstract

With improved survival, more patients with prior breast cancer are at risk of having a second primary cancer diagnosed. The pattern and impact of second primary cancers following breast cancer is important for overall breast cancer therapeutic management. Our study is a first analysis of the trend of second primary tumours over time in terms of incidence, sites with significantly elevated risks and correlation with stage, molecular subtype and therapeutic strategies conducted in Eastern Europe in postmenopausal women with breast cancer. Patients and methods: Our study population included 28 patients with prior breast cancer (BC) and second primary tumours, which were diagnosed and treated in our Institution between 2004 and 2017. The criteria for selection were based on the completeness of the documentation of the first treatment for breast cancer, stage of disease, molecular subtype, the site of origin of the second tumours and the survival data. Results: An increased risk of second primary cancer was associated with the 51–60 years age group (53.6%), with the greater prevalence in patients living in urban environments (82.1%). The use of chemotherapy increased the risk of the occurrence of gynecological second malignancies (75%). Our study is a first analysis of the trend of second primary tumours over time in terms of identifying sites with significantly elevated risks and correlation with therapeutic strategies conducted in Eastern Europe in postmenopausal women with breast cancer. Conclusions: Our study is a first analysis of the trend of second primary tumours over time in terms of correlation with luminal subtype and stage at diagnosis of primary cancer sites with significantly elevated risks and correlation with therapeutic strategies in postmenopausal women with breast cancer conducted in Eastern Europe. The reported time from primary to second primary malignancy onset, with a significantly higher rate for postmenopausal breast cancer patients, was less than one year (50%). With the advances and wider availability of genetic testing (e.g., gene panels), patients diagnosed with multiple primaries should be increasingly investigated for an underlying cancer predisposition. Postmenopausal women with breast cancer may benefit from increased surveillance and advice to avoid second malignancies.

## 1. Introduction

Breast cancer is a group of malignancies counting more than 500,000 cases at the European level, being by far the most frequently diagnosed neoplasm (28.2% of the total) [1]. With significant disparities across Western and Eastern countries, it is accountable as the fourth most frequent cause of death among all types of cancer. In an analysis per-formed by Autier and colleagues, it is outlined the fact that in some Western countries, BC mortality decreased in recent years by ≥20%, while in Central and Eastern European countries, mortality did not decline or even increased. Such is the case of Romania, which encountered a remarkable increase of 17% in BC mortality [2]. Indeed, a more recent analysis on trends and predictions in BC mortality in Europe shows that Romania, Poland, Ukraine and the Russian Federation also have unfavourable trends compared to Western countries and the UK [3].

Significant treatment advances have led to increases in the overall survival rate in patients with BC. However, BC survivors have significantly increased risks for second cancers at different sites, due to shared etiology, host, environmental and lifestyle factors, genetic pre-disposition but also to primary BC treatment [4,5]. Second primary cancer (SPC) may occur months or years after the first cancer was diagnosed. Therefore, the pattern and impact of second primary cancers following BC are important for overall BC therapeutic management.

The current criteria used for defining multiple primary tumours are different from one study to another [6]. The two main definitions are provided by the Surveillance Epidemiology and End Results (SEER) and the International Association of Cancer Registries and the International Agency for Research on Cancer (IACR/IARC). SEER considers histology, site, laterality and time since initial diagnosis to identify SPCs, while the IARC/IACR rules are more exclusive: Only one tumour is registered for an organ, irrespective of time, unless there are histological differences. For this reason, contralateral BC is considered as a single tumour as per the IACR/IARC definition, while the SEER considers contralateral BC as an SPC [5]. For this study, we used the SEER guidelines and included contralateral BC as an SPC.

The incidence of SPC in patients with prior BC has been studied in case series and cohort studies using population-based cancer registries. However, most reports were from Western Europe, Scandinavia and Asia [4,7,8,9,10,11,12,13]. As such, the patterns of secondary primary cancers’ development in the Eastern European population are still poorly understood.

Currently, BC represents a major public health problem in Romania. Low awareness of the disease risk, the lack of a national screening program and late-stage diagnosis of the disease explain this. To date, no scientific data is covering the incidence of SPCs in the Romanian population with BC or other types of cancer. We initiated a preliminary study focused on the epidemiological and clinicopathological characteristics of postmenopausal women with prior BC [14]. Using our institutional database, in the present study, we intend to investigate the incidence, survival and patterns of SPCs, including demographic data, stage at diagnosis, molecular subtype and concomitant therapies prescribed for the primary BC, and in this way, we collect data for a bigger trial and maybe the prediction of the time on onset.

SPC is not only a particularly difficult event for patients but could also be an important prognostic factor among cancer survivors [12,15]. For these patients, the challenge is to find the best therapeutic strategy that addresses both cancers, without increased toxicity. It is noted that unlike patients with single tumours, these patients are often excluded from clinical trials and thus have more limited therapeutic options. Moreover, previous studies have suggested that breast cancer treatment could play a crucial role in the development of other primary malignancies as a late effect and consequence of the treatment [5,11]. Furthermore, SPC is one of the most serious and life-threatening adverse effects following radiotherapy, experienced by the growing number of cancer survivors worldwide, according to multiple studies, with a particular interest for postmenopausal women [16,17,18,19].

## 2. Materials and Methods

This study was a retrospective, observational, single institution study, performed in a general Oncology specialized Outpatient Unit.

### 2.1. Patients

In a previous study conducted in our Institution, we investigated the medical records of 1000 patients registered in our database to identify postmenopausal females with a primary diagnosis of breast cancer as their first malignancy, admitted in our Institution between May 2004 and December 2017 [14]. Among these, a total of 271 female patients had premenopausal BC (27.1%), 721 female patients had postmenopausal BC (72.1%) and only 8 patients were males (0.8%).

Out of this postmenopausal BC population, during November–December 2020, we looked at additional collected data and identified some 40 female subjects with double malignancies. Based on the other cancer date of diagnosis, we excluded 12 patients that were diagnosed with other neoplasms before BC. Twenty-eight patients were selected for further analysis. This selection was also based on the completeness of documentation of the first treatment for BC, stage, molecular subtype, the site of origin of the second tumours and the survival data.

Different types of interventions for the treatment of primary BC included surgery (26 patients), chemotherapy (21 patients), hormonotherapy (24 patients), radiotherapy (15 patients) and the use of anti-vascular endothelial growth factor (VEGF) agents (3 patients).

The Institutional Review Board (IRB) of the ONCOMED Outpatient Unit approved the former retrospective study in July 2017. The retrospective data were collected and reviewed in compliance with the ethical standards set out by the IRB and with the Declaration of Helsinki. For this retrospective study, the patients’ consent for the review of their medical records was not required by the Steering Committee of the Institution. As we consider the present study an ancillary study to further explore the characteristics of females with postmenopausal BC, additional approval from the IRB was not deemed necessary.

### 2.2. Data Collection

Data were collected with the support of the ONCOMED Outpatient Unit.

We used the Surveillance, Epidemiology and End Results (SEER) Guidelines to define second malignancies and the St. Gallen 2017 consensus for the definition of molecular subtype [20].

According to the SEER definition of multiple primary tumours, the following criteria were used in our study: (1) tumours with ICD-O-3 histology codes that are different at the first, second or third number are multiple primaries; (2) one tumour characterized as “adeno-carcinoma, NOS” and another as a specific adenocarcinoma is regarded as a single tumour; (3) an invasive tumour following an in situ tumour >60 days after diagnosis is multiple primaries; (4) tumours with ICD-O-3 topography codes that are different at the second and/or third characters are multiple primaries; (5) tumours diagnosed >1 year apart are multiple primaries.

The population registry included 28 eligible patients with prior BC and second malignancies. All personal data pertaining to the study population were anonymized, and an IRB approved its use.

The demographic data that we considered for this study were: age, defined as the age at the time of diagnosis, and living environment (rural or urban).

The clinical data collected included the stage of the disease at the time of diagnosis. The clinical data recorded in the Oncology outpatient charts and the surgery medical letters were also used as a source of documentation. TNM classifications, both clinically and/or pathologically, were used for establishing the stage of the disease. For the patients who underwent surgical treatment, the pathological TNM was used. For the remaining patients, we used the clinical TNM. Depending on the year of diagnosis, we used the 5th, 6th and 7th AJCC TNM classification [21,22].

The molecular subtype was classified for invasive tumours according to the 2017 St. Gallen Consensus, depending on the ER, PR, KI-67 and HER2 status. According to this classification, patients were divided into five subgroups: luminal A, luminal B, luminal HER2-positive, non-luminal HER2-positive and triple-negative breast cancer [20].

These parameters were obtained from the immunohistochemistry (IHC) examination of the breast cancer tissue sample. The tissue was examined in 3 local laboratories based in Timisoara, Romania. All these labs used manual or automatic platforms for the IHC results. Estrogen Receptor (ER) and/or Progesterone Receptor (PR) were considered positive when the value was >1%. The cut-off value for cell proliferation associated antigen (KI-67) to define luminal A to luminal B tumours was considered to be 20%. To distinguish HER2-positive from HER2-negative tumours, pathologists performed IHC and in situ hybridization tests and use the ASCO/CAP guidelines valid at the time of the examination.

### 2.3. Statistical Analysis

The statistical analysis was performed using the Statistical Software for Social Sciences (SPSS version 22, IBM^®^, Armonk, NY, USA). For the number of subjects studied, we used descriptive statistical methods in tabular and graphic representation.

We used the Frequency Analysis to analyse the measures of central tendency, dispersion and percentiles. This was useful to highlight the number of subjects with different SPC sites but also to highlight the various therapeutic interventions, as well as their effects over time. The results of the applied treatments were reflected in the survival rate of the patients in our study.

## 3. Results

Second primary tumours occurred in 28 out of 721 (3.8%) postmenopausal patients with BC, with a significant percentage of malignancies occurring in the contralateral breast sites (25.0%), gynecologic sites (21.3%) and gastrointestinal sites (14.3%), as shown in Table 1.

**Figure 1:**
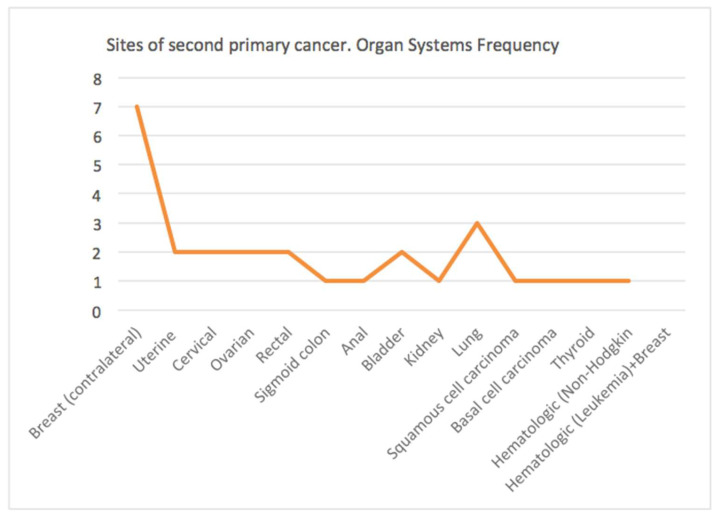

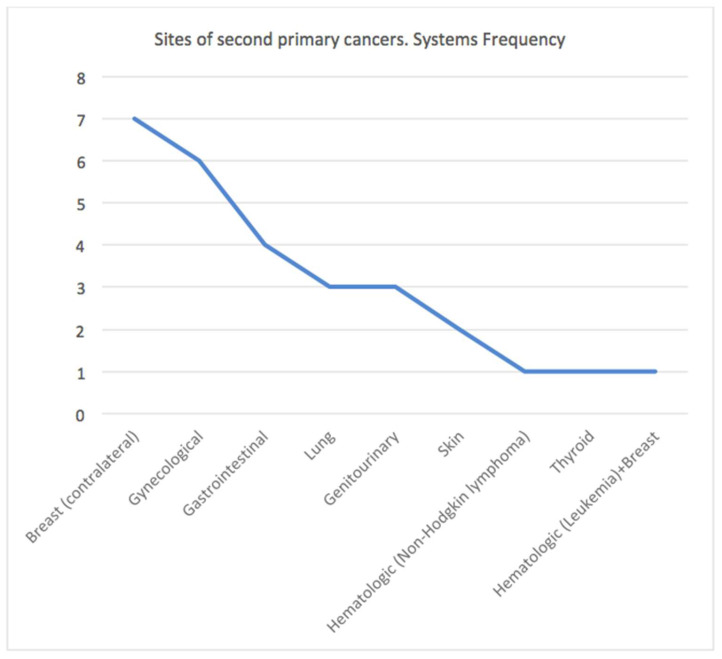
Sites of secondary primary cancers divided into organ systems and organ frequency.

As reported below, over the study period, there was an increase in the prevalence of the contralateral breast second cancer (seven cases), followed by lung cancer (three cases), gynecological (two cases with cervical cancer and two cases with ovarian cancer) and gastrointestinal cancers (two cases with rectal cancer and one case with sigmoid colon cancer). These data are graphically represented in Figure 1.

**Figure 2:**
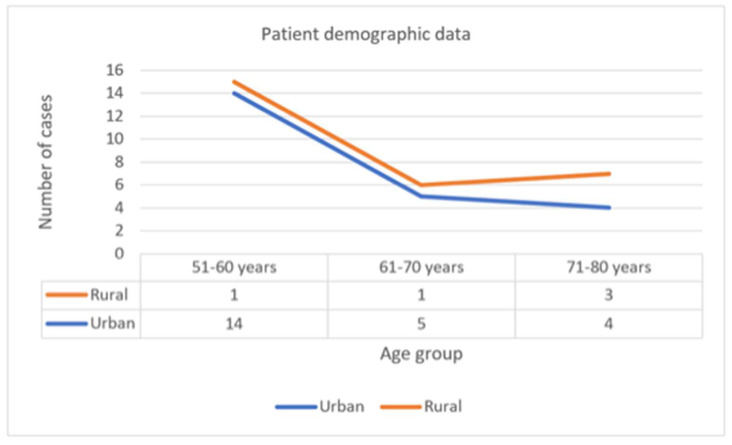
Patient demographic data.

The analysed demographic data included different age groups (51–60, 61–70 and 71–80 years, respectively) and the living environment (rural and urban). Out of 28 patients, the distribution showed prevalence for urban settings (*N* = 23; 82.1%) and a small group (*N* = 5; 17.9 %) were from rural settings. The most prevalent age group was 51–60 years from urban settings (93.3%), as shown in Figure 2.

**Figure 3:**
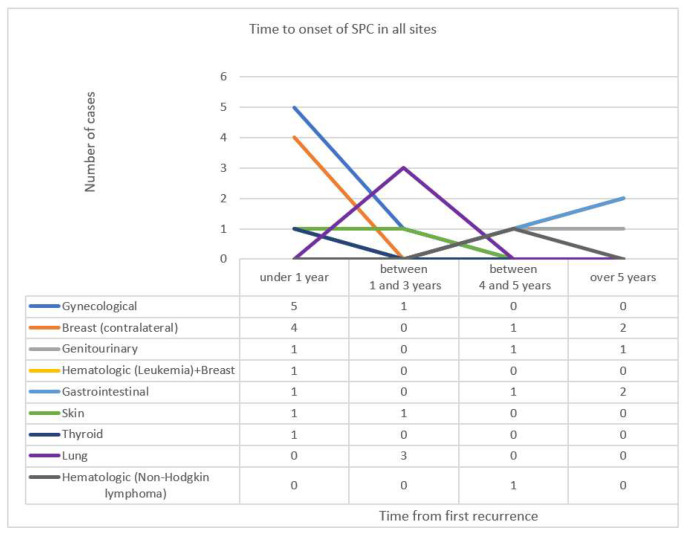
Time from diagnosis of primary breast cancer to second primary malignancies in all sites.

We analysed the time from the diagnosis of the primary breast cancer to that of second malignancies, divided by four interval groups, as follows: less than 1 year, between 1 and 3 years, between 4 and 5 years and more than 5 years. There were no SPC cases reported to occur in the interval of 3–4 years in our population. The most prevalent interval for SPC occurrence was under 1 year, and the most prevalent cancers sites reported were gynecological sites (five cases), followed by contralateral breast cancers (four cases), followed by gastrointestinal sites. These results are summarized in Figure 3.

**Figure 4:**
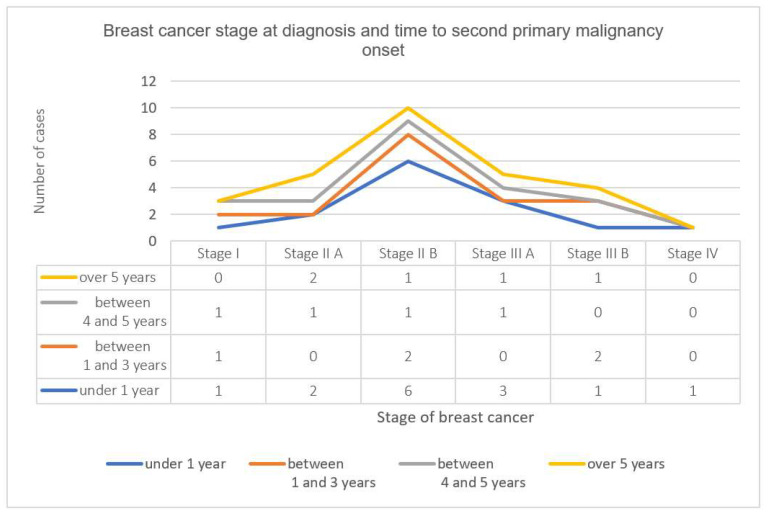
Breast cancer stage at diagnosis and time to second primary malignancy onset.

Long-term survival with multiple primaries is variable and is influenced by cancer type and stage at diagnosis. We performed a correlation between BC stage at diagnosis and time to second primary malignancy onset. As shown in Figure 4, we found a lower incidence of second primaries in stage I BC, with a better prognosis and shorter occurrence period for stage IIB (six cases within a year after the BC diagnosis) and stage IIIA (three cases within a year after the BC diagnosis).

Out of 28 patients, the most prevalent molecular subtype found was to be Luminal B (*N* = 12), followed by Luminal A (*N* = 11). In Luminal B patients, the onset of second primary malignancy occurred in less than a year (*N* = 6; 6.50%), followed by 1–3 years (*N* = 3; 3.25%) and more than 5 years (*N* = 2; 16.7%). We had no cases of triple-negative/basal-like breast cancer in our population. The results are summarized in Table 2. There were no SPC cases reported to occur in the interval of 3–4 years in our population.

Different therapeutic interventions for the treatment of primary BC are presented in Table 3, Table 4, Table 5 and Table 6.

For only 7.14% of patients (*N* = 2), surgery was not performed. For the remaining 26 patients, Table 3 shows the timeframe to BC comparing different SPC sites.

Our data shows that 46.15% of the patients (*N* = 12) that underwent surgical intervention for the primary BC had a registration of an SPC within less than 1 year, 19.23% (*N* = 5) had an SPC within the period of 1–3 years, 15.38% (*N* = 4) had an SPC within the period of 4–5 years and 19.23% (*N* = 5) after 5 years.

The late toxic effects of radiotherapy and chemotherapy also contribute to the increased risk for a second primary tumours after breast cancer. We analysed the timeframe to SBC comparing different SPC sites for 21 patients receiving chemotherapy for primary BC.

We analysed the timeframe to SPC, comparing different second primary cancer sites, for 15 patients receiving radiotherapy. Our results show that subsequent second malignancies of the breast, gynecological, genitourinary and leukemia may occur within less than after treatment with radiotherapy.

The use of VEGF agents for the treatment was limited to three patients. The analysis of time to SPC occurrence shows variations depending on the cancer site. We found one site in the contralateral breast with a period of more than 5 years to second malignancy, one genitourinary site (with a period of 4–5 years) and one lung site (with a period of occurrence of 1–3 years).

Out of 28 patients, a total of 24 patients used hormone therapy drugs for the treatment of primary BC, as shown in Table 6.

Table 6 shows a correlation between SPC sites, SPC onset and hormonotherapy used for the treatment of primary BC. Contralateral breast SPC and gynecological sites (number of cases: six out of six) are the most prevalent in terms of their occurrence, with faster occurrence within one year after the primary BC diagnosis. Secondly, gastrointestinal sites are reported to occur but within a longer period of time (total number of cases: four, with two cases occurring after more than 5 years after primary BC diagnosis).

**Figure 5:**
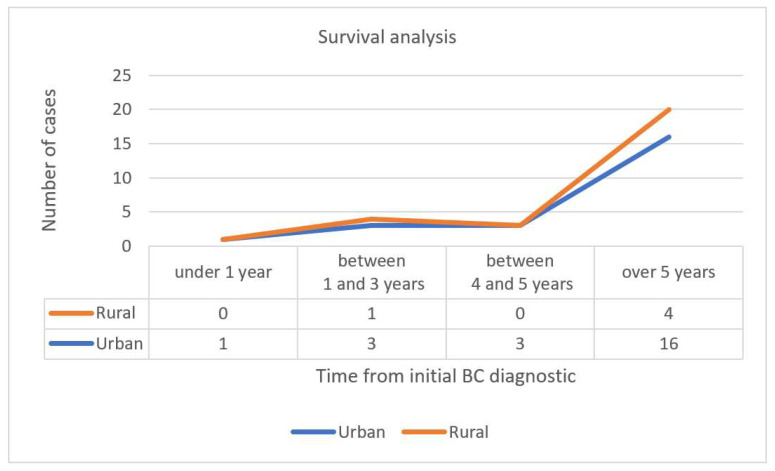
Survival analysis of living environment of the studied population.

Our survival analysis performed for the studied population included a correlation with the living environment. The data shows that SPC after BC have a relatively high survival, with 71.42% patients being alive after 5 years. The demographic distribution of surviving patients indicated that a better prognostic is correlated with the urban living environment (16 patients in urban areas vs. 4 patients in rural areas) (Figure 5).

## 4. Discussion

Across the literature, data concerning the incidence rates and the SPC sites vary substantially. For example, Young reported a 4.1% incidence rate for second primary malignant tumours, while Li reported a cumulative incidence of 7.43% at 10 years, 14.41% at 15 years and 20.08% at 20 years [7,23]. Similar to Young, we report in our study a 3.88% incidence rate for SPC. The difference can result from including only postmenopausal females in our study.

In a large Spanish cohort study, Molina-Montes reported a median incidence rate of only 1.39% incidence for all cancer sites, with the most frequents sites being skin, non-melanoma (4.27), endometrium (3.04) and kidney (2.20) [24]. Young found that the most common second double primary malignancies sites were thyroid, stomach, endometrium, cervix and lung, while Andersson found a modest increase in the risk of second cancers in early-stage BC, with the most affected sites being potentially irradiated sites, such as the lung [4,7]. The risk for cancer of corpus uteri was increased for patients treated with tamoxifen while the risk of second ovarian cancer was increased in younger patients. The same finding was reported by Molina-Montes [24], with endometrial cancer being the most frequent type of SPC in women over 50 years.

Our study found that a higher risk to detect SPC in postmenopausal BC females is posed by contralateral breast (25%) and gynecological sites (21.3%). These sites are frequently identified within one year after the BC diagnosis.

However, with a larger number of patients surviving for longer times after treatment, we emphasize the fact that the incidence but also the pattern of SPC after BC observed after different therapeutic interventions have changed, with 71.42% of patients being alive after 5 years.

This is the first institution-based investigation among BC survivors in Romania, providing data on SPCs. Moreover, our study combined the trends in incidence of site-specific SPCs with molecular subtypes, initial stages and treatment strategies for primary BC.

However, the present study has several limitations. First, it should be noted that this study has a relatively small sample size, and our data are limited to Caucasian, postmenopausal females. Therefore, a larger study including a more diverse sample size could be needed to extend the conclusions from the present study. Second, data regarding other contributing risk factors for cancer, such as the family history of cancer and reproductive factors of the patients were not available. As such, a detailed description and comparison between these factors and SPC risk could be the subject for further investigations.

## 5. Conclusions

Our study is a first analysis of the evolution of second primary tumours over time, in terms of correlation with luminal subtype and stage at diagnosis of primary cancer sites with significantly elevated risks and correlation with therapeutic strategies in postmenopausal women with BC, conducted in Eastern Europe.

The most common SPC in this study population was contralateral breast cancer, which occurred in seven patients (25.0%), followed by malignancies reported in gynecological sites cancer in six patients (21.4%) and gastrointestinal sites in four patients (14.3%). The reported time from primary BC to second primary malignancy onset, with a significantly higher rate for postmenopausal BC patients, was less than one year (50%).

When examining the time to diagnosis of second primary neoplasm among females with BC, our study showed that in the vast majority of patients, this interval was less than one year. Regarding the history of therapeutic interventions for BC, our results showed that regardless of the therapy administered (surgery, radiotherapy, chemotherapy, hormonotherapy or anti-VEGF treatment), the SPC onset was under one year after the BC diagnosis. This result, together with the SPC incidence of more than 80% for patients living in urban environments suggests the hypothesis of the genetic and environmental component in the etiology of SPC rather than the side effects of antineoplastic therapy, which usually occur after a long time after the start of treatment.

Continued surveillance is needed to detect SPCs and to provide improved survival rates in patients with BC. Moreover, it is important to conduct further investigations after the first BC diagnosis, especially in the contralateral breast. As standard CT scans may sometimes be unable to detect new lesions, with the increasing use of more sophisticated imaging methods, namely positron-emission tomography-computed tomography (PET-CT) and whole-body magnetic resonance imaging (MRI), it is not uncommon to find suspicious second primary lesions. With the advances and wider availability of genetic testing (e.g., gene panels), patients diagnosed with multiple primaries should be increasingly investigated for an underlying cancer predisposition. The gain in knowledge on patients with hereditary cancer and cancer survivors will hopefully allow for the development of specific management and surveillance measures.

## Figures and Tables

**Table 1:** Sites of second primary cancers.

Second Primary Cancers	Organ Systems Frequency (*N*)	Organ Frequency (*N*)	Percent, %
Breast (contralateral)	7	7	25.0
Gynecological	6		21.3
Uterine		2	7.1
Cervical		2	7.1
Ovarian		2	7.1
Gastrointestinal	4		14.3
Rectal		2	7.1
Sigmoid colon		1	3.6
Anal		1	3.6
Genitourinary	3		10.7
Bladder		2	7.1
Kidney		1	3.6
Lung	3	3	10.7
Skin	2		7.2
Squamous cell carcinoma		1	3.6
Basal cell carcinoma		1	3.6
Thyroid	1	1	3.6
Hematologic (Non-Hodgkin lymphoma)	1	1	3.6
Other type of cancers combined Hematologic (Leukemia) + Breast	1	1	3.6
Total	28	28	100

**Table 2:** Correlation between luminal subtype and time to second primary malignancy onset.

Molecular Subtype	Patients, *N*	Time to Second Primary Malignancy			
		˂1 Year	1–3 Years	4–5 Years	≥5 Years
Luminal A	11	6	1	2	2
Luminal B	12	6	3	1	2
Luminal HER positive	3	1	0	1	1
Non-luminal HER positive	2	1	1	0	0
Total	28	14	5	4	5

**Table 3:** Correlation between SPC onset and surgery performed on primary BC.

Surgery Performed for Primary BC	Patients, *N*	Time to Second Primary Malignancy			
		˂1 Year	1–3 Years	4–5 Years	≥5 Years
Breast (contralateral)	7	4	0	1	2
Gynecological	5	4	1	0	0
Gastrointestinal	4	1	0	1	2
Genitourinary	3	1	0	1	1
Lung	3	0	3	0	0
Skin	1	0	1	0	0
Thyroid	1	1	0	0	0
Other type of cancers combined Hematologic (Leukemia) + Breast	2	1	0	1	0
Total	26	12	5	4	5

**Table 4:** Correlation between SPC onset and chemotherapy performed on primary BC.

Chemotherapy in Primary BC	Patients, *N*	Time to Second Primary Malignancy			
		˂1 Year	1–3 Years	4–5 Years	≥5 Years
Breast (contralateral)	6	3	0	1	2
Gynecological	5	4	1	0	0
Gastrointestinal	2	0	0	0	2
Genitourinary	3	1	0	1	1
Lung	3	0	3	0	0
Skin	1	0	1	0	0
Hematologic (Non-Hodgkin lymphoma)	1	0	0	1	0
Total	21	8	5	3	5

**Table 5:** Correlation between SPC onset and radiotherapy performed on primary BC.

Radiotherapy in Primary BC	Patients, *N*	Time to Second Primary Malignancy			
		˂1 Year	1–3 Years	4–5 Years	≥5 Years
Breast (contralateral)	5	3	0	0	2
Gynecological	4	4	0	0	0
Gastrointestinal	3	1	0	1	1
Genitourinary	2	1	0	0	1
Other type of cancers combined Hematologic (Leukemia) + Breast	1	1	0	0	0
Total	15	10	0	1	4

**Table 6:** Correlation between SPC onset and hormonotherapy performed on primary BC.

Hormonotherapy	Patients, *N*	Time to Second Primary Malignancy			
		˂1 Year	1–3 Years	4–5 Years	≥5 Years
Breast (contralateral)	6	4	0	0	2
Gynecological	6	5	1	0	0
Gastrointestinal	4	1	0	1	2
Genitourinary	3	1	0	1	1
Lung	1	0	1	0	0
Skin	2	1	1	0	0
Thyroid	1	1	0	0	0
Hematologic (Non-Hodgkin lymphoma)	1	0	0	1	0
Total	24	13	3	3	5

## Data Availability

The data that support the findings of this study are available from the corresponding author upon reasonable request.

## Abbreviations

BC	Breast cancer
SPC	Second Primary Cancer
SERR	Surveillance Epidemiology and End Results
IACR	International Association of Cancer Registries
IARC	International Agency for Research on Cancer
VEGF	Vascular Endothelial Growth Factor
IRB	Institutional Review Board
TNM	Tumour (T), Nodes (N), Metastases (M)
AJCC	American Joint Committee on Cancer
IHC	Immunohistochemistry
ER	Estrogen receptor
PR	Progesterone receptor
KI-67	A cell proliferation-associated antigen
PET-CT	Positron-Emission Tomography-Computed Tomograph
MRI	Magnetic Resonance Imaging
